# Mixed Clinical Pictures of Endogenous Endophthalmitis in a Relapse Leukemic Patient

**DOI:** 10.7759/cureus.49167

**Published:** 2023-11-21

**Authors:** Annuar Z Azmi, Sylves Patrick, Mohamad Israk B Isa, Shuaibah Ab. Ghani

**Affiliations:** 1 Department of Pediatric Ophthalmology, Sabah Women and Children Hospital, Kota Kinabalu, MYS; 2 Department of Ophthalmology, University Malaysia Sabah, Kota Kinabalu, MYS; 3 Department of Ophthalmology, Universiti Malaysia Sabah, Kota Kinabalu, MYS

**Keywords:** endophthalmitis, acute myeloid leukaemia, relapse, leukaemia, endogenous endophthalmitis

## Abstract

Endogenous endophthalmitis is rare but sight-threatening in leukemic patients, which can have devastating sequelae. We report a case of a 15-year-old teenager with acute myeloid leukemia on relapse, presented with a mixed picture of endogenous endophthalmitis. The diagnosis dilemma in this patient proved difficult as Investigations and management can be challenging as young teenagers are usually less cooperative than adults. Endogenous endophthalmitis is not uncommon in this group of patients; however, mixed clinical pictures are almost unheard of, and the final diagnosis can be misleading if not treated accordingly. Viral infections such as cytomegalovirus (CMV), bacterial, and fungal are all considered potential opportunistic infections. Diagnosis of endogenous endophthalmitis is complex and relies heavily on the clinical characteristics of each organism supported by intravitreal tapping and culture samples. However, data from endogenous endophthalmitis in leukemic patients is scarce nowadays across the board. In this case report, we highlight the challenges of managing endogenous endophthalmitis in a young leukemic patient due for bone marrow transplantation. Future studies are needed to investigate the current microorganism trends and treatments available. An algorithm for managing endophthalmitis in immunosuppressed patients should be done to provide a better approach from the get-go.

## Introduction

Patients with immunosuppressive conditions such as leukemia are prone to a get secondary infection [1]. Those receiving chemotherapy had a higher risk of infection due to reduced cell-mediated immunity [2]. Endogenous endophthalmitis is a possible infection that may cause debilitating outcomes in this group of patients [3]. In younger children and teenagers, getting a diagnosis seems treacherous as the patient may be uncooperative, and a thorough examination has to be done under general anesthesia. Viral, bacterial, and fungal causes are known culprits for opportunistic infection in those with weakened immune systems [1]. Subsequent treatment may be challenging as patients may require multiple intravitreal medications depending on the isolated organisms.

## Case presentation

A 15-year-old girl with underlying relapsed acute myeloid leukemia (AML) presented with left eye blurring of vision associated with eye pain for four days before being referred for ocular screening. She was diagnosed with AML in November 2020. The patient needed urgent treatment and clearance from the ophthalmology team as she was due for a bone marrow transplant in June 2021. However, in June 2021, she had a relapsed AML, and another cycle of chemotherapy was planned for her. On day nine of her second cycle of chemotherapy, she developed neutropenic fever and was covered with intravenous IV meropenem (40 mg/kg/day). During this time, she developed generalized body rashes, and IV amphotericin (1 mg/kg/day) was commenced to cover for fungal skin candidiasis. Septic workup from blood and urine was unremarkable for bacterial and fungal growth. Shortly after, she developed the aforementioned eye symptoms in early July 2021. The blurring of vision was gradual in onset and not associated with scotoma. No floaters or photophobia were noted. The eye pain was mild and generalized, with no precipitating factors that aggravated the pain.

**Figure 1:**
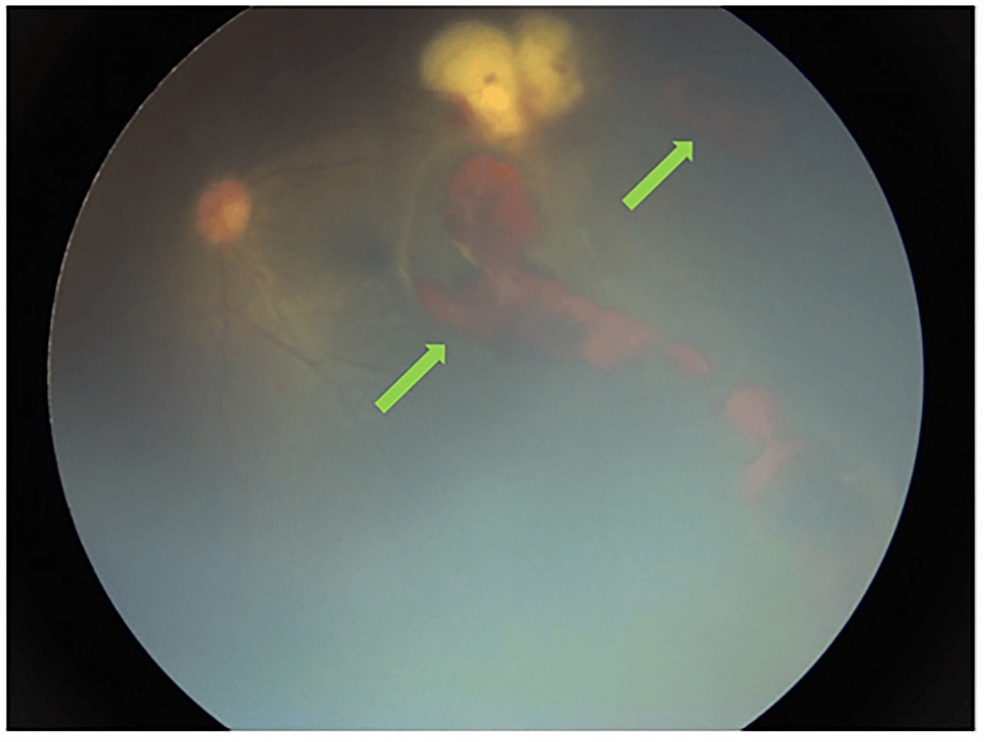
Dilated fundus examination of the LE revealed extensive pre-retinal and intra-retinal hemorrhages (green arrow) LE - left eye

**Figure 2:**
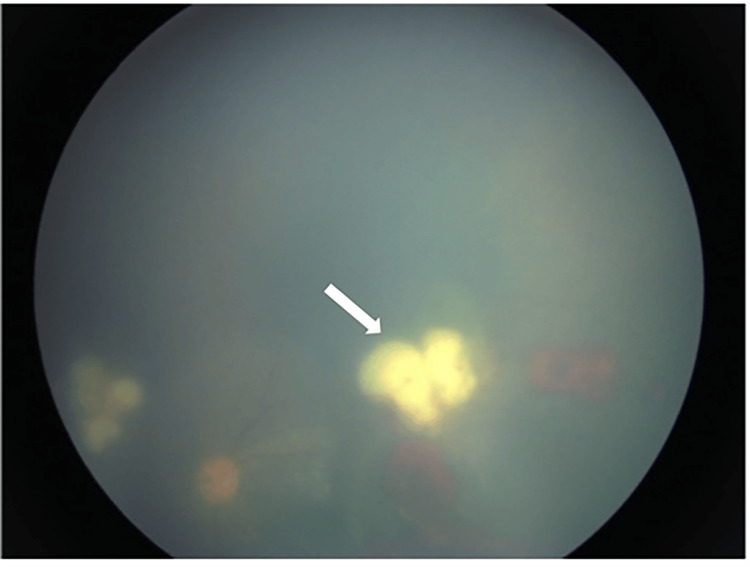
Retinitis (white arrow) at the superotemporal arcade next to the optic disc

**Figure 3:**
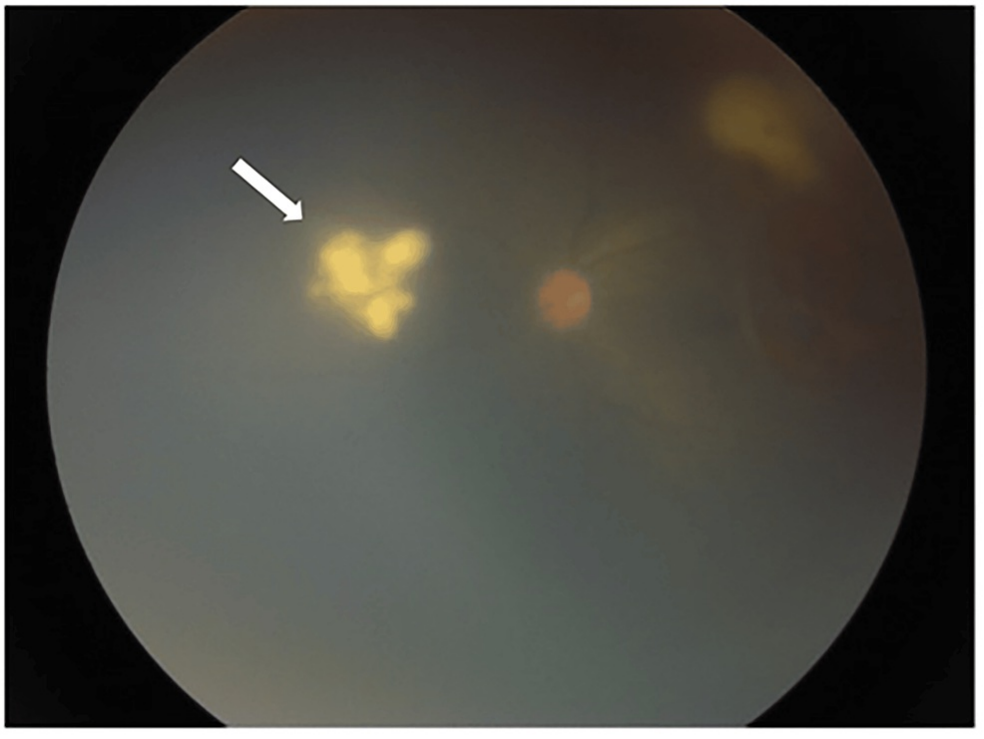
Retinitis can be seen nasal to the optic disc (white arrow)

Upon examination, the best corrected visual acuity (BCVA) was 6/9 in the right eye (RE) and 6/60 in the left eye (LE). No relative afferent pupillary defect (RAPD) was noted. LE anterior segment examination revealed anterior uveitis with 2+ cells in the anterior segment (AC)-otherwise, neither keratoprecipitates (KP), iris nodule, nor hypopyon noted in the anterior chamber. Intraocular pressure was normal for both eyes. Dilated fundus examination of the LE revealed extensive pre-retinal and intra-retinal hemorrhages (Figure 1), extending one disc diameter (DD) from the fovea to the far temporal. The view was hazy due to cells in the anterior chamber. Perivascular sheating can be seen over the temporal aspect of the bleeding. Retinitis can be seen at the superotemporal arcade (Figure 2) and nasally (Figure 3) next to the optic disc. The optic disc was hyperemic and had no vitritis during the initial assessment. Examination of the RE anterior and posterior segment was otherwise unremarkable.

**Figure 4:**
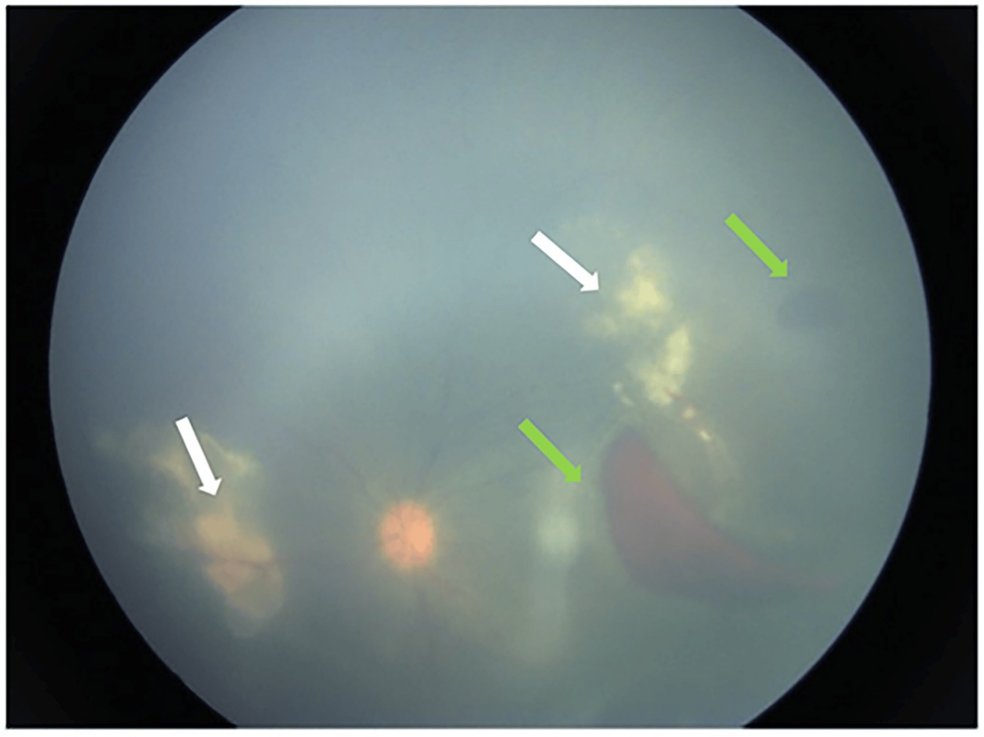
Contracting hemorrhages (green arrow) and retinitis (white arrow)

**Figure 5:**
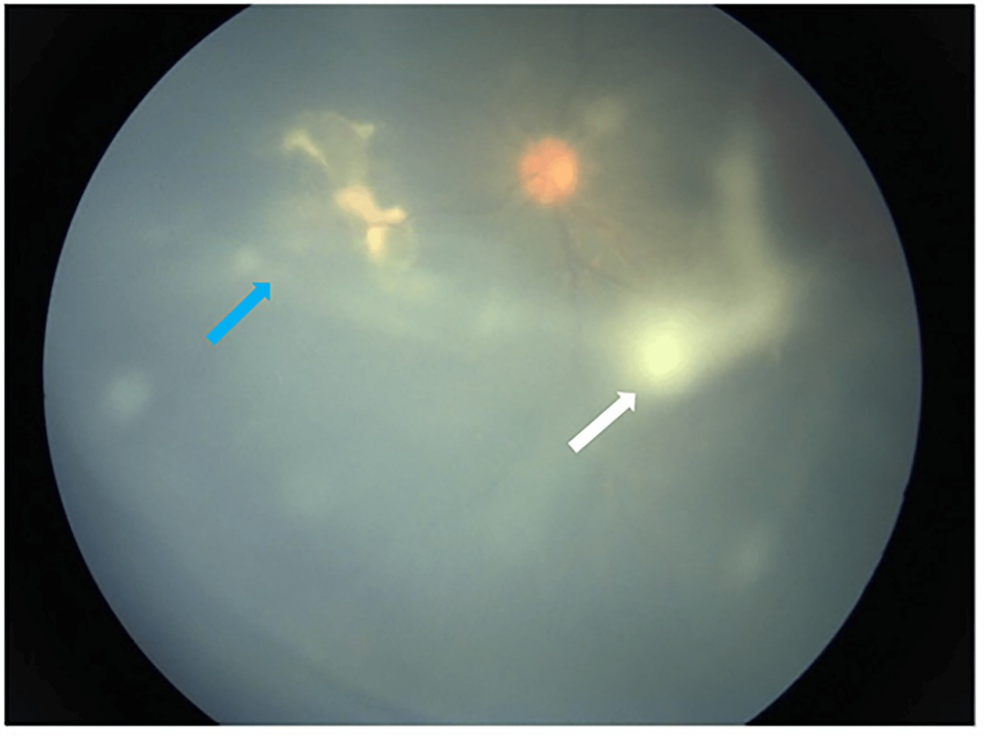
Localized vitritis surrounding the nasal retinitis (blue arrow), with a suspicious string of pearls forming anterior to the macula region (white arrow)

**Figure 6:**
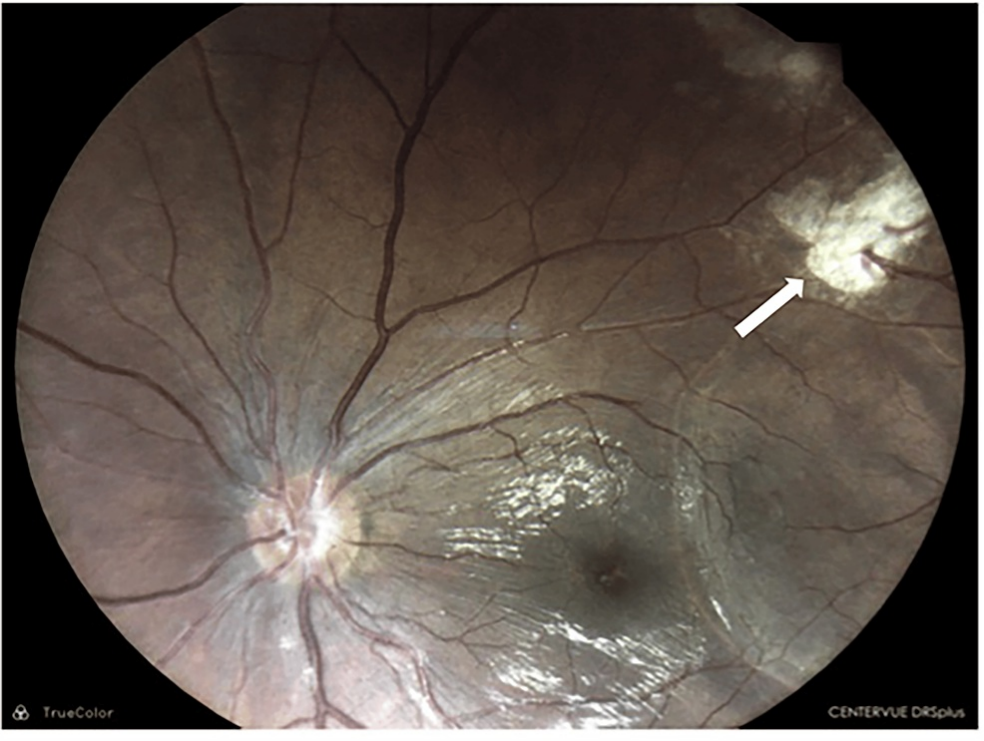
Following the TPPV, the areas of hemorrhages and vitritis resolved, and the retinitis area (white arrow) became flatter TPPV - trans-pars planar vitrectomy

The initial impression was LE CMV retinitis, and the patient was treated with IV ganciclovir (10 mg/kg/day) for two weeks. Examination under anesthesia (EUA) was subsequently planned with an intravitreal (IVT) tap and IVT ganciclovir (2mg in 0.05ml) injection. IVT tap result revealed coagulase-negative staphylococci (CONS), which is sensitive to Vancomycin. Otherwise, the vitreous sample for fungal, viral studies, and cytology was negative. The case was further discussed with a medical retina specialist, and the patient was commenced with IVT vancomycin (2mg in 0.1ml) every 72 hours until marked clinical improvement. Initially, the patient showed a response to treatment by the evidence of contracting hemorrhages and retinitis area (Figure 4). Localized vitritis surrounding the nasal retinitis was noted, with a suspicious string of pearls forming anterior to the macula region (Figure 5) during the eighth EUA. Therefore, IVT amphotericin B (5mcg in 0.1ml) was given in addition to IVT vancomycin. Subsequent EUA shows the retinitis became more well-defined and flatter; however, the pearl of strings remained. IVT voriconazole (2.5mcg in 0.1ml) was added and given during the subsequent EUA, and vitreoretinal (VR) opinion was sought. After discussion with the VR team, trans-pars planar vitrectomy (TPPV) was planned for this patient with IV Voriconazole(8mg/kg/day) for two weeks. All this while the areas of hemorrhage maintained 1 DD away from the fovea, and her vision never dropped below 6/60. Following the TPPV, the areas of hemorrhages and Vitritis resolved, and the retinitis area became flatter (Figure 6), replaced with what remained of scars. BCVA improved to 6/12 for the LE. Following endophthalmitis's resolution, the patient underwent a bone marrow transplant in February 2023.

## Discussion

Endophthalmitis is a sight-threatening condition and needs urgent treatment. Since leukemic patients are immunocompromised, patients with underlying Leukaemia possess higher risks of developing endophthalmitis. Frequent chemotherapy will lead to bone marrow suppression and increase exposure to opportunistic infections [4]. One retrospective study of 271 patients for endogenous endophthalmitis found one patient with a background of immunocompromised secondary to Leukaemia [5]. Patients with Leukaemia are prone to opportunistic infection because of the nature of the disease coupled with chemotherapy [6]. A variety of infections in the form of viral, bacterial, and fungal can attack the immunocompromised patient [7].

For this patient, the initial presentation mimicked a CMV retinitis infection with the pattern of hemorrhagic retinitis. The diagnosis was initially made by clinical judgment as the pattern matches CMV presentation. CMV is one of the most common viral that can infect immunocompromised patients [8]. The initial management was to commence treatment with intravitreal ganciclovir and IV Ganciclovir for two weeks. IVT tapping for this patient revealed coagulase-negative staphylococcus (CoNS), which shows sensitivity to vancomycin, and therefore, the diagnosis and management were changed to endogenous bacterial endophthalmitis. Zhang et al., in their series of endophthalmitis cases, highlight that the coagulase-negative staphylococcus group is one of the top two common isolates alongside the Streptococcus group [5]. CoNS are part of the average human skin commensal and have low virulence, but they can cause severe infections, especially in immunocompromised patients [9].

After initiation of intravitreal vancomycin, the patient was showing improvement in the area of retinitis contracting; however, during the eighth EUA, we noted the appearance of strings of pearl that raised the question of whether this patient had a co-infection with fungal endophthalmitis considering that patient was treated for fungal skin rashes during her neutropenic fever episode. Due to the clinical pictures resembling fungi at the later stage of the disease, we give the benefit of no doubt and commence treatment with IVT voriconazole and systemic anti-fungal despite no fungal growth isolated from the IVT tapping.

The prevalence of fungal endophthalmitis is lower than bacterial endophthalmitis and is a clinical diagnosis that may be supported by vitreous culture [10]. It is also possible for the patient to have more than one infection as she is immunosuppressed, as evidenced by early bacterial culture isolated from the initial IVT tap and fungal pictures in the later stage of the disease. This case is the first presentation of endophthalmitis in leukemic patients reported in a literature review with mixed clinical pictures.

In our case, the decision not to do PPV earlier is because the area of involvement never involved the central fovea, and the patient's LE vision never dropped beyond 6/60; therefore, we committed to the IVT vancomycin injection as it was initially improved until the appearance of the string of pearls that may be signifying overlapping fungal infection in the later stage of the disease. Thus, we refer to a vitreoretinal (VR) surgeon for intervention, as fungal endophthalmitis is more virulent and associated with poorer outcomes [11].

## Conclusions

Patients with endogenous endophthalmitis who are immunosuppressed can be challenging to treat, especially with mixed clinical pictures. Co-infection with multiple organisms is a possibility due to the weakened immune system. It is time to develop guidelines for endogenous endophthalmitis, especially in leukemic patients. When in doubt, pars plana vitrectomy can be a savior.
